# The effect of estrogen and its receptors on the progression of cervical intraepithelial neoplasia in postmenopausal women through synergistic interaction with HPV

**DOI:** 10.1186/s12905-026-04460-9

**Published:** 2026-04-10

**Authors:** Haifeng Liu, Yanfang He, Wenpan Geng, Yanan Huang, Huanhuan Gao, Yingying Zhai, Zhaoxia Wang

**Affiliations:** 1 Gynecology, The First Clinical Medical College, Shanxi Medical University, No. 56, Xinjian South Road, Yingze District, Taiyuan City, Shanxi Province 030001 China; 2 Academy of Medical Sciences, Shanxi Medical University, No. 56, Xinjian South Road, Yingze District, Taiyuan City, Shanxi Province 030001 China; 3 Gynecology, The First Hospital of Shanxi Medical University, No. 85, Jiefang South Road, Yingze District, Taiyuan City, Shanxi Province 030001 China

**Keywords:** Estrogen, Estrogen Receptor, Menopausal Window/Non-Window Period, Cervical Intraepithelial Neoplasia

## Abstract

**Objective:**

To observe the progression of cervical intraepithelial neoplasia (CIN) in Human papillomavirus (HPV)-positive women receiving vaginal local estrogen therapy during the postmenopausal window period versus the non-window period, and to analyze differences in CIN progression across different stages of menopause. To compare differences in estrogen receptor (ER) expression across different postmenopausal stages and during the progression of CIN. To clarify that the use of local estrogen in postmenopausal women requires careful evaluation of benefits versus risks to avoid indiscriminate medication. To provide scientific evidence for clinical decision-making regarding estrogen use at different postmenopausal stages and for the prevention and control of CIN.

**Methods:**

The study population consisted of postmenopausal women who were HPV-positive and had a first-time colposcopy diagnosis of cervicitis or CIN1. Follow-up examinations were conducted 6 months to 2 years later, with local vaginal estrogen application prior to follow-up. Participants were divided into a “window period” group and a “non-window period” group based on the timing of menopause, and colposcopy results were recorded and compared between the two groups. Additionally, normal cervical epithelial tissue samples were independently collected from both time periods. Cervical tissue samples from cases of cervicitis, CIN1, CIN2, and CIN3 were collected and categorized into cervicitis/CIN1 and CIN2/CIN3 groups. All specimens underwent ER immunohistochemistry, and differences in ER expression were compared across groups.

**Results:**

The window-period group included 44 participants, and the non-window-period group included 41 participants. Follow-up results showed that 7 patients (15.9%) in the window period group had CIN2 or higher, while 15 patients (36.6%) in the non-window period group had CIN2 or higher. Compared with the window period group, the progression rate was significantly higher in the non-window period group, with a statistically significant difference (*P* < 0.05). Ten cervical tissue samples were collected from each of the window period and non-window period groups, for a total of 20 samples. There were 5 cases each in the cervical inflammation, CIN1, CIN2, and CIN3 groups, totaling 20 cases. Immunohistochemical results indicated that ER levels in cervical epithelium were significantly higher in the window period group than in the non-window period group, with a statistically significant difference (*P* < 0.05). ER levels decreased significantly during the progression from cervical inflammation/CIN1 to CIN2/CIN3, and the difference was statistically significant (*P* < 0.05).

**Conclusion:**

Estrogen and its receptors interact synergistically with HPV to influence CIN progression; compared to the window period, estrogen use during the non-window period may further accelerate CIN progression.

## Introduction

The incidence and mortality rates of cervical cancer are declining globally, easing the overall disease burden. However, in economically underdeveloped countries and regions, cervical cancer incidence and mortality rates remain persistently high. This phenomenon highlights disparities in cervical cancer prevention and control efforts across different nations and regions [1]. Cervical cancer is the most common malignant tumor of the reproductive system among Chinese women [2]. As the only preventable malignant tumor currently, cervical cancer screening technology has made significant progress. With the widespread adoption of cervical cancer screening, CIN—a precancerous lesion—has gradually gained public recognition. The primary cause of CIN is persistent infection with high-risk human papillomavirus (hR-HPV) [3]. Research data indicate that the incidence risk of HPV-related diseases is projected to show an upward trend over the next 15 years [1]. Therefore, the issue of cervical lesions caused by HPV infection still warrants high-priority attention.

CIN, as a disease of the female reproductive system, is likely closely associated with estrogen in its development. Estrogen possesses immunomodulatory effects, and relevant studies indicate that low-dose vaginal estrogen therapy is effective for treating postmenopausal urogenital syndrome [4]. Estrogen use enhances local immune responses in the vagina and reduces pathological damage to the vaginal tissue [5]. Conversely, other studies indicate that estrogen promotes cervical carcinogenesis. During the early stages of HPV infection, estrogen increases viral load, exacerbates viral persistence, and worsens the severity of cervical lesions [6, 7]. Estrogen exerts its effects through ER, which mediates multiple signaling pathways that promote cervical lesions [8, 9]. This demonstrates the dual action of estrogen.

Menopause refers to the permanent cessation of ovarian function, leading to symptoms associated with estrogen deficiency. Menopausal hormone therapy (MHT) is widely used to treat various clinical symptoms in postmenopausal women [10]. The menopausal window period refers to the decade following menopause, while the non-window period refers to the decade after menopause and beyond [4]. Multiple studies have shown [11, 12], that MHT use during the postmenopausal window period often yields greater benefits, but research on this phenomenon in cervical lesions remains largely unexplored. Against this backdrop, this study examines the differential impact of estrogen use on cervical lesion progression between postmenopausal window and non-window populations. It analyzes changes in ER expression levels in cervical tissue from the postmenopausal window to the non-window phase, further exploring the dynamic shifts in ER expression during the progression from inflammatory/CIN1-grade tissue to CIN2/CIN3-grade lesions.

## Methods

### Study population

All subjects included in this study were approved by the Ethics Committee of the First Hospital of Shanxi Medical University.

#### Clinical data

Patients who visited the Outpatient Colposcopy Unit of the Department of Gynecology at the First Hospital of Shanxi Medical University between January 2021 and April 2025 and met the following inclusion and exclusion criteria were selected.

Inclusion Criteria: Postmenopausal women (amenorrhea ≥ 12 months); history of local vaginal estrogen use, defined as “window period” (< 10 years postmenopause) or “non-window period” (≥ 10 years postmenopause); history of two colposcopy examinations with the first showing cervicitis or CIN1; complete clinical follow-up records; voluntary participation and signed informed consent. The specific regimen for estrogen is as follows: Proestren cream, 10 mg per application, administered vaginally once daily for 7 to 21 consecutive days.

Exclusion Criteria: Premenopausal or perimenopausal women; History of hysterectomy or cervical resection; History of HPV vaccination; History of estrogen use other than vaginal estrogen (e.g., hormone therapy after oophorectomy); History of smoking; Severe systemic diseases: cardiac, hepatic, or renal insufficiency (e.g., severe heart failure, acute coronary syndrome, severe arrhythmias, or myocardial infarction within the past 3 months; liver failure, decompensated cirrhosis, or severe coagulation disorders; acute respiratory failure, severe pulmonary infection, or acute exacerbation of severe chronic obstructive pulmonary disease); systemic infectious diseases; patients with incomplete clinical data; and those who refuse to participate.

#### Specimen data

Cervical tissue specimens were collected separately. Inclusion criteria were: women who had reached natural menopause; a clear pathological diagnosis of cervical tissue; no history of local vaginal estrogen therapy; and patients who had signed an informed consent form. Exclusion criteria were: patients with concomitant cervical conditions (such as cervical polyps or cysts), malignant tumors, or incomplete clinical data; and those who refused to participate.

To ensure balanced and comparable groups, matching was performed based on age, duration of menopause, and severity of cervical lesions. Age and duration of menopause are important factors influencing estrogen receptor expression in cervical tissue, while lesion severity directly affects the risk of disease progression. Matching these factors reduces confounding bias and enhances the reliability of the results.

The specific groups are as follows: the group of normal cervical tissue from the postmenopausal window period and the group of normal cervical tissue from the non-window period of menopause; the group of inflamed cervical tissue from the postmenopausal window period; and the groups of CIN1, CIN2, and CIN3 tissue.

### Research methods

#### Clinical data

Clinical data were collected via a questionnaire, including: age at menopause; number of pregnancies and deliveries; results of two HPV tests, two Thinprep Cytology Tests (TCT), and two colposcopies; and details regarding the use of local vaginal estrogen following the first colposcopy, including duration of use and age at initiation. Newly enrolled participants were classified into the postmenopausal window CIN1 group and the non-postmenopausal window CIN1 group. The study examined whether there were differences in the progression of CIN lesions at the second colposcopy between the two groups.

#### Specimen data

The collected specimens were subjected to immunohistochemical analysis to assess ER expression patterns. All specimens were fixed in 10% neutral formalin, followed by routine dehydration and paraffin embedding. Paraffin sections 4 μm thick were prepared and subjected to hematoxylin and eosin (HE) staining and immunohistochemical staining. The ER primary antibody was purchased from Shanghai Gene Technology Co., Ltd. (Catalog No.: GT205602). This antibody is a ready-to-use working solution that requires no dilution and can be applied directly.

For the postmenopausal and perimenopausal groups, four 40X fields of epithelial tissue were photographed from each slide. Cervicitis and CIN1 were grouped together, and CIN2 and CIN3 were grouped together; four 40× fields of epithelial tissue were photographed from each slide. Changes in the number of ERs in the epithelial tissue were compared. Specimen data were analyzed using ImageJ to evaluate the immunohistochemical results of the cervical epithelium. Optical density measurements were performed on the brown-stained areas (ER staining) in the images. The following parameters were selected for measurement: area, mean gray value, integrated density, area fraction, and limit to threshold.

### Statistical analysis

Statistical analysis was performed using SPSS 27.0. Descriptive analysis of clinical data focused on continuous variables. Parametric tests were conducted for continuous variables. Variables following a normal distribution were expressed as mean ± standard deviation, while those not following a normal distribution were expressed as median (interquartile range). Independent samples t-tests were used for between-group comparisons of continuous variables. Categorical variables were analyzed using the chi-square test. Multivariate analysis was performed using binary logistic regression. For cervical tissue specimens, the mean and standard deviation were calculated for a set of histological slides. Independent-samples t-tests were used to verify and analyze differences in ER expression between the window period group and the non-window period group, as well as between the cervicitis/CIN1 group and the CIN2/CIN3 group. *P* < 0.05 was considered statistically significant.

## Results

The population of clinical data meeting the inclusion criteria consisted of 68 participants in the menopausal window group and 60 participants in the non-menopausal window group, for a total of 128 participants. The final population included in the analysis (after excluding participants who were lost to follow-up or had incomplete data) comprised 44 participants in the menopausal window group and 41 participants in the non-menopausal window group, for a total of 85 participants.

Tissue samples meeting inclusion criteria: 10 cases in the window period group and 10 cases in the non-window period group, for a total of 20 cases. There were 5 cases each of cervicitis, CIN1, CIN2, and CIN3, totaling 20 cases, for a combined total of 40 cases.

### Basic situation analysis

**Fig. 1:**
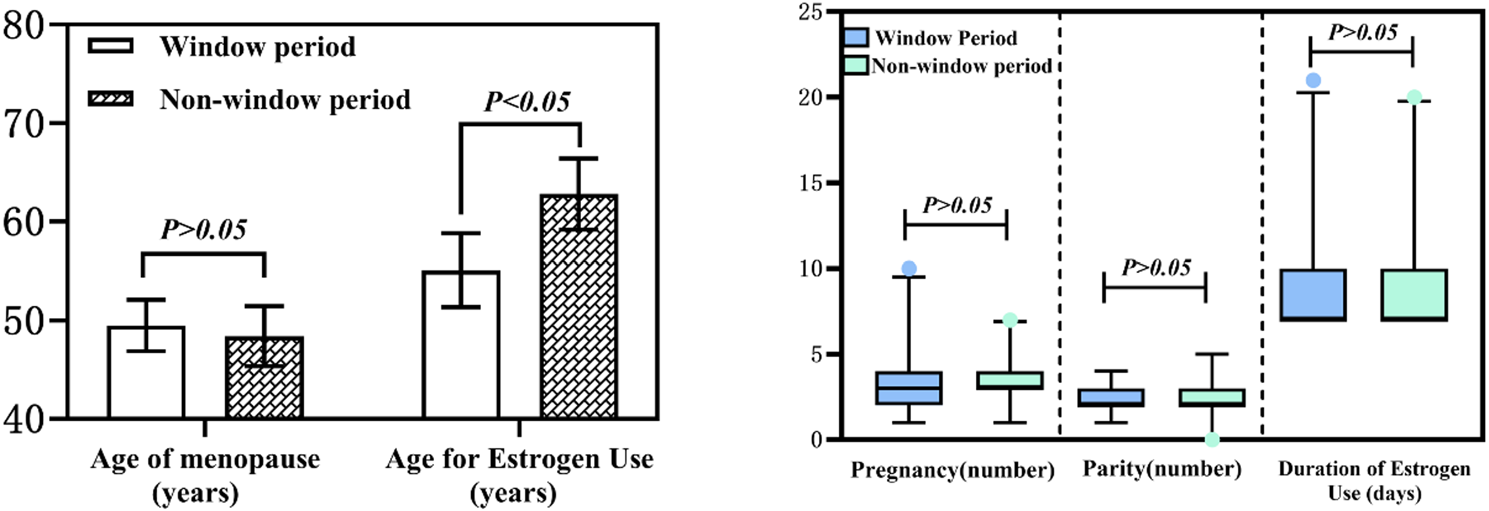
Basic characteristics of the two groups

Normality tests were conducted for parity, number of births, age at menopause, age at estrogen initiation, and duration of estrogen use (days). Age at menopause and age at estrogen initiation followed normal distributions across all groups. Independent samples t-tests were used to analyze differences between groups. Pregnancy history, parity, and duration of estrogen use failed to meet normality criteria; therefore, nonparametric tests were used to analyze differences between groups (Fig. 1). Results indicated no statistically significant differences between groups for parity, number of births, age at menopause, or duration of estrogen use (*P* > 0.05). However, a statistically significant difference was observed in age at estrogen initiation (*P* < 0.05), confirming baseline comparability between groups. Detailed statistical results are presented in Table 1.

**Table 1:** Analysis of the basic characteristics of the two groups

	xˉ±s/ *P*_50_(*P*_25_~*P*_75_)		F/Z	*P*
	Window period	Non-window period		
Age of menopause (years)	49.50 ± 2.610	48.41 ± 3.041	0.675	0.081
Age for estrogen use (years)*	55.09 ± 3.747	62.80 ± 3.614	0.026	< 0.01
Pregnancy (number)^#^	3(2 ~ 4)	3(3 ~ 4)	-0.325	0.745
Parity (number)^#^	2(2 ~ 3)	2(2 ~ 3)	-1.005	0.315
Duration of estrogen use (days)^#^	7(7 ~ 10)	7(7 ~ 10)	-0.742	0.458

* indicates *P* < 0.05

^#^ indicates non-normal distribution as determined by normality testing

### Window period/non-window period univariate analysis

**Fig. 2:**
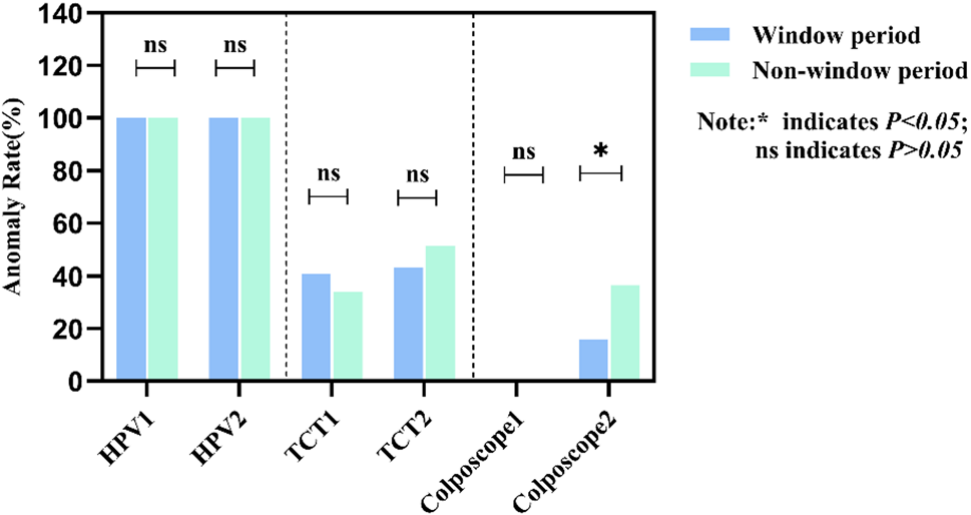
Cross-sectional Analysis of Window Period vs. Non-Window Period

**Fig. 3:**
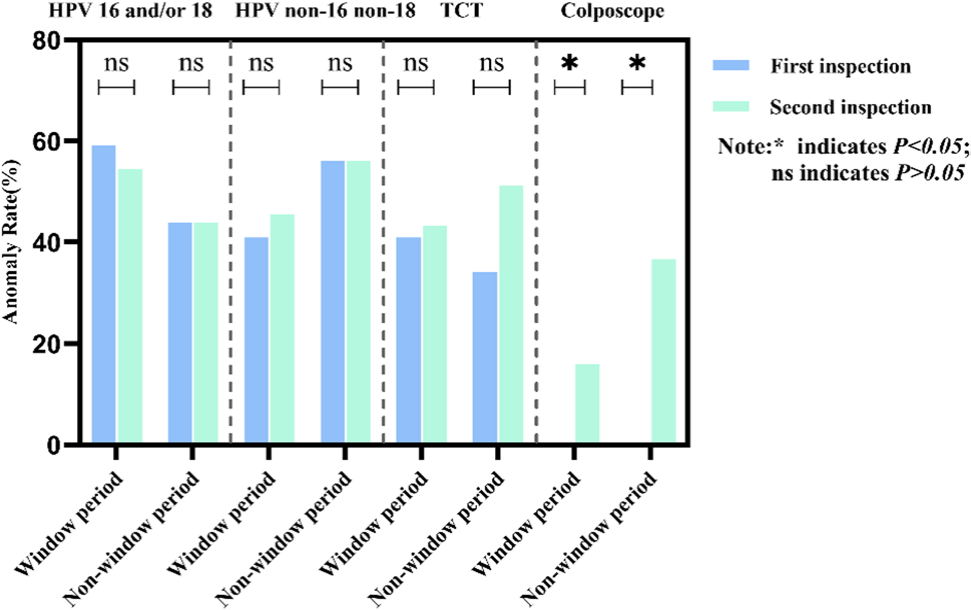
Longitudinal Analysis of the Window Period vs. Non-Window Period

HPV, TCT, and colposcopy results were compared between the window period and non-window period cohorts from the first and second examinations. Both cohorts showed a 100.0% hR-HPV positivity rate. HPV test results were categorized into HPV16 and/or HPV18 infection and non-HPV16 and non-HPV18 infection. TCT results were categorized as non-NILM or Negative for Intraepithelial Lesion or Malignancy (NILM), where non-NILM included Atypical Squamous Cells of Undetermined Significance (ASCUS), Atypical Squamous Cells, Cannot Exclude High-Grade Squamous Intraepithelial Lesion (ASC-H), Low-grade Squamous Intraepithelial Lesion (LSIL), and High-grade Squamous Intraepithelial Lesion (HSIL). Colposcopy findings were classified as ≥CIN2 or < CIN2. All data were categorical variables. Cross-tabulation chi-square tests were used to analyze differences between groups (Fig. 2). Progression in HPV, TCT, and colposcopy results between the first and second visits was compared between the window period and non-window period cohorts. HPV progression was defined as non-HPV16 and non-HPV18 infection at the first visit followed by HPV16 and/or HPV18 infection at the second visit. Progression in TCT was defined as NILM at baseline and non-NILM at follow-up. Progression in colposcopy was defined as < CIN2 at baseline and ≥CIN2 at follow-up. All data were categorical variables. Paired chi-square tests assessed HPV and TCT progression, while Fisher’s exact test evaluated colposcopy progression (Fig. 3). Results showed no statistically significant differences in HPV or TCT outcomes between window period and non-window period cohorts (*P* > 0.05). However, colposcopy progression rates differed significantly between groups, with the non-window period showing significantly higher progression rates than the window period (*P* < 0.05) (Table 2).

**Table 2:** Comparative analysis of differences in HPV, TCT, and colposcopy findings

		Window period (*n* = 44)	Non-window period (*n* = 41)	χ^2^	*P*
HPV16 and/or 18	First time	26(59.1%)	18(43.9%)	1.961	0.161
	Second time	24(54.5%)	18(43.9%)	0.962	0.327
Abnormal TCT	First time	18(40.9%)	14(34.1%)	0.414	0.520
	Second time	19(43.2%)	21(51.2%)	0.550	0.458
Abnormal colposcopy*	First time	0(0.0%)	0(0.0%)		
	Second time	7(15.9%)	15(36.6%)	4.730	0.030

* indicates *P* < 0.05

### Multivariate analysis of window period vs. non-window period

**Fig. 4:**
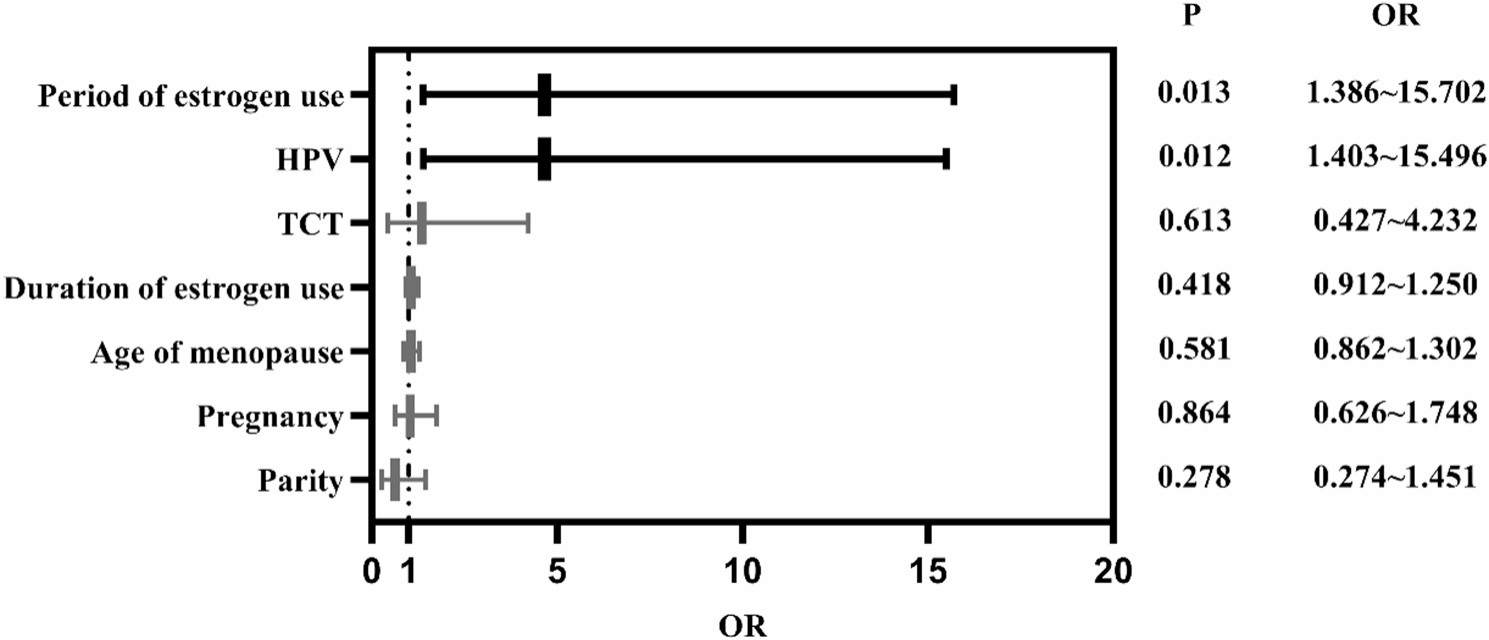
Forest plot of factors influencing the progression of CIN after menopause

Multivariate logistic regression analysis included estrogen use period, HPV results, TCT results, duration of estrogen use, age at menopause, parity, and number of births (Fig. 4). Results showed that the risk of estrogen use during the postmenopausal non-window period was 4.665 times higher than that during the postmenopausal window period (95% CI 1.386–15.702, *P* = 0.013 < 0.05). The risk of cervical lesions caused by HPV16 and/or HPV18 infection during the non-window period after menopause was 4.663 times higher than that of non-16, non-18 h-HPV infection (95% CI 1.403–15.496, *P* = 0.012 < 0.05).

### Comparison of ER results between window period and non-window period

**Fig. 5:**
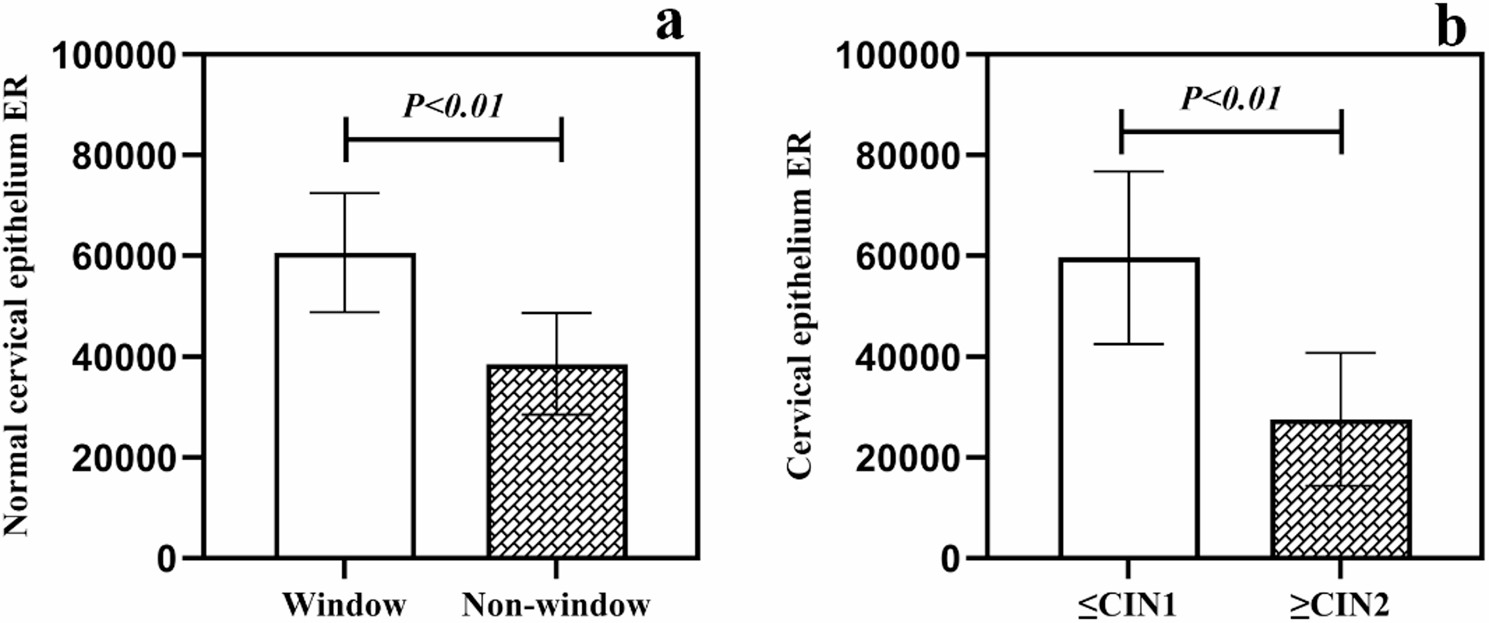
Analysis of Differences in ER Expression Levels in Cervical Epithelial Tissue

**Fig. 6:**
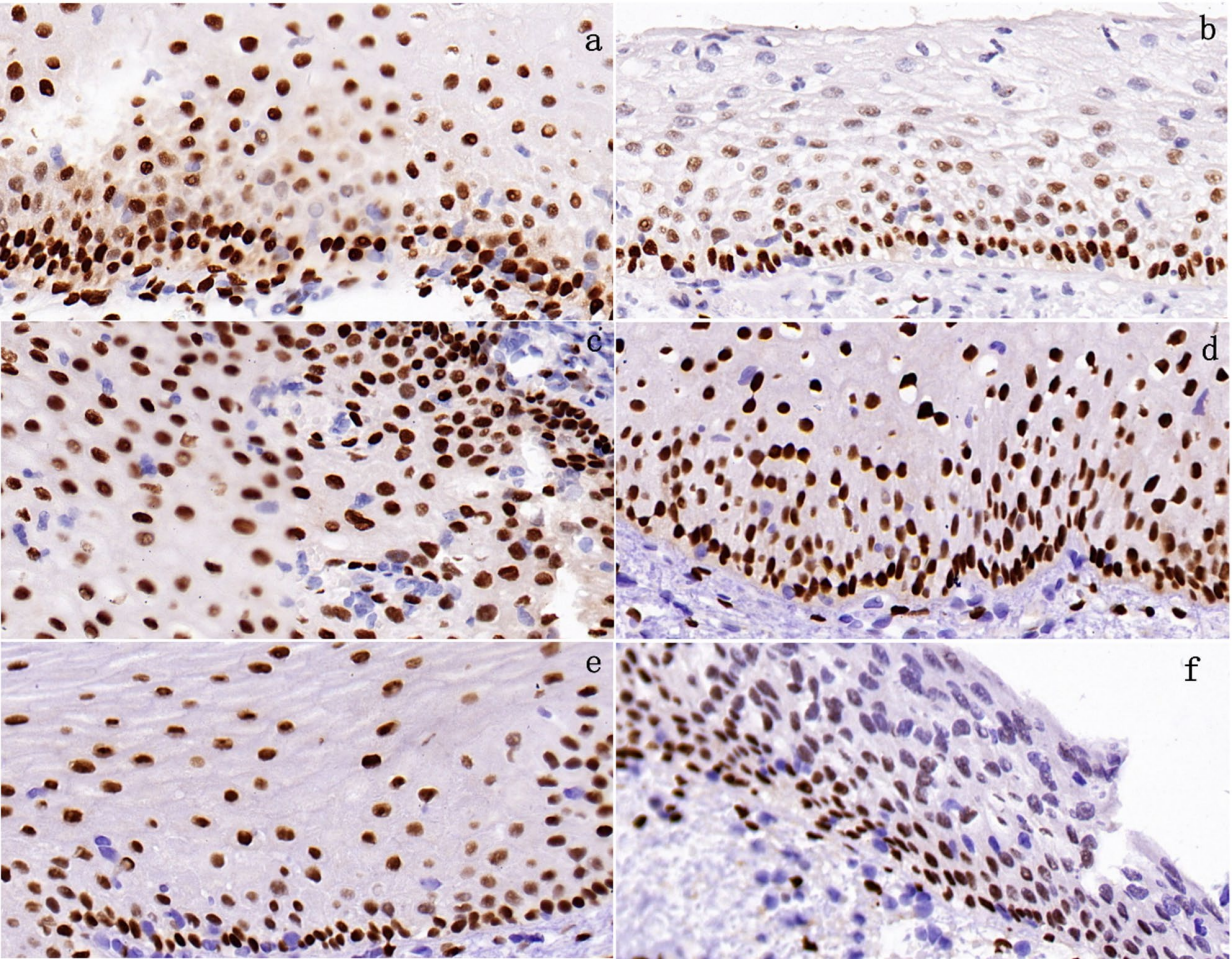
Are all 40X photographs. **a**: Normal tissue during the menopausal window period. **b**: Normal tissue outside the menopausal window period. **c**: Cervicitis. **d**: CIN1. **e**: CIN2. **f**: CIN3

ER immunohistochemical results revealed that the number of ER-positive cervical epithelial cells in both the menopausal window period group and the non-window period group followed a normal distribution. Further comparison of ER counts between the two groups via an independent samples t-test yielded T = 8.985, *P* < 0.05. indicating that the ER count in cervical epithelial tissue during the window period was significantly higher than that during the non-window period, with a statistically significant difference (Figs. 5a and 6a-b). Detailed results are presented in Table 3.

### Analysis of ER change trends during CIN progression

To further validate the level of ER count changes during CIN progression, the window period cohort was divided into two groups. The first group included 5 cases each of window period cervicitis and CIN grade 1, while the second group comprised 5 cases each of CIN2 and CIN3. Immunohistochemical analysis revealed that ER counts in cervical epithelial tissues from both groups followed a normal distribution. Independent samples t-tests comparing ER counts between groups yielded the following results: T = 9.381, *P* < 0.05. indicating a significantly higher ER count in cervical epithelial tissue from the cervicitis/CIN1 group compared to the CIN2/CIN3 group (statistically significant; Figs. 5b and 6c-e).

**Table 3:** Differential analysis of ER groups

Grouping	*N*	mean	Standard deviation	Standard error	T	*P*
Window period	10	60633.03	11862.48	1875.62	8.985	< 0.001
Non-window period	10	38575.20	10017.53	1583.91		
Cervicitis/CIN1	10	59673.40	17100.94	2703.90	9.381	< 0.001
CIN2/CIN3	10	27606.05	13225.92	2091.20		

## Discussion

### implications of findings

This study reveals that vaginal estrogen use at different time points influences CIN progression in postmenopausal women. Specifically, the progression rate for CIN occurring outside the window period (36.6%) was significantly higher than that within the window period (15.9%). This suggests that estrogen use during specific time windows may yield differing benefits for postmenopausal women. Furthermore, immunohistochemical analysis revealed a consistent decline in ER levels from the window period to the non-window period, and from cervical inflammation/CIN1 to CIN2/CIN3. These findings support the critical role of estrogen and its receptors in CIN progression.

### Differences in estrogen receptors during the window period and non-window period

As the population ages, the number of postmenopausal women continues to rise, making attention to this demographic a significant social issue. This study indicates that ER numbers gradually decrease with age, potentially limiting estrogen’s effects through estrogen receptors. During the window period, high ER numbers enable physiological estrogen doses to exert stronger immunoprotective effects locally on the cervix. In contrast, during the non-window period, the low ER count means that physiological doses of estrogen may place this population in a relatively high-estrogen state. Consequently, multiple ER signaling pathways may promote the progression of cervical lesions.

Previous studies have shown that in vaginal tissue, ER expression is higher in the premenopausal period than in the perimenopausal period, while ER expression in the perimenopausal period is higher than in the postmenopausal period [13]. Another experiment on the cardiovascular system revealed that ER expression was significantly higher in the early postmenopausal stage compared to the late postmenopausal stage [14]. Due to limited reports on the differential levels of ER expression in the cervix during the early and late stages of menopause, this study addresses a gap in the field. Findings indicate that ER expression levels are higher during the menopausal window period compared to the non-menopausal window period. Further analysis reveals that within the same timeframe (specifically the menopausal window period in this study), ER expression decreases progressively with the advancement of CIN.

### Differences in estrogen levels during the postmenopausal window and non-window periods

Postmenopausal women gradually lose their sensitivity to estrogen responses with increasing age. Follicle-stimulating hormone (FSH) and luteinizing hormone (LH) levels in postmenopausal women remain stable during the postmenopausal window period. However, over time, FSH levels exhibit a gradual downward trend [15]. During early menopause, estrogen levels fluctuate significantly. Due to declining hormone levels, the hypothalamus exhibits a pro-ovulatory effect, with hypothalamic neurons expressing estrogen receptors undergoing hypertrophy [16]. Research indicates that compared to women in early menopause, those in late menopause exhibit reduced sensitivity to gonadotropin-releasing hormone (GnRH) stimulation of the hypothalamus [15]. Therefore, whether it be estrogen or estrogen receptor levels, sensitivity to estrogen remains high during the menopausal window phase. Consequently, cervical tissue may exhibit heightened responsiveness to estrogen replacement therapy during this period, potentially yielding superior local immune effects.

### Exploring the mechanism of estrogen-HPV synergy

Persistent HPV infection can induce abnormal gene expression in host cells, increasing cancer risk. Simultaneously, HPV promotes tissue angiogenesis to deliver increased oxygen supply, further facilitating tumor growth [17]. HPV induces excessive proliferation of epithelial cells while simultaneously impairing the immune function of keratinocytes through multiple pathways, leading to immune dysfunction [18]. Estrogen synergizes with this effect; estrogen treatment elevates levels of pro-inflammatory factors in HPV E6/E7 cells, with certain factors also showing an upward trend in cervical cancer cells. This suggests that estrogen may exacerbate cervical inflammation by regulating pro-inflammatory factors, thereby promoting the occurrence and progression of cervical cancer [19]. HPV infects cervical keratinocytes and disrupts their normal differentiation process. Furthermore, HPV suppresses the host’s innate immune response, thereby creating favorable conditions for viral replication [20]. Estrogen plays a crucial role in the physiological functions of the cervix. Recent studies indicate that the mechanism by which estrogen acts on cervical keratinocytes remains unclear. Notably, in skin-related research, steroid hormones are closely associated with various inflammatory diseases [21]. Among these, estrogen can influence changes in skin thickness by promoting the proliferative activity of keratinocytes [22]. These findings further support the existence of a synergistic interaction between estrogen and HPV, which may jointly regulate the proliferation process of cervical epithelial cells.

### Research limitations and future directions

Due to the limited research period, the sample size was constrained. In future studies, we plan to expand the sample size to enhance the representativeness of the research. Building upon this foundation, we aim to further explore the mechanisms by which estrogen mediates immune effects and activates ER-related signaling pathways to promote CIN progression. Since the study did not include individuals who had received the HPV vaccine, its findings may provide a reference for assessing the safety of vaginal estrogen use in postmenopausal women. Therefore, future studies are advised to include HPV vaccination status as an evaluation criterion.

## Conclusion

Studies have shown that there is a specific “window period” effect when postmenopausal women take estrogen supplements: compared with the window period, taking estrogen supplements outside the window period may increase the risk of CIN progression. At the same time, ER levels in cervical epithelium were significantly higher during the window period than outside it. Further research found that, over the same period, ER levels in cervical epithelium gradually decreased as cervical lesions progressed. Through clinical observations and experimental analyses, this study provides evidence regarding the association between the timing of postmenopausal estrogen use and the risk of CIN. The results indicate that the use of vaginal estrogen—at the dosage, duration, and type specified in this study—during the non-window period after menopause may be associated with an increased risk of CIN progression. This suggests that clinicians should pay attention to the potential impact of estrogen use at different time points on the risk of cervical lesions. When prescribing estrogen for patients outside the window period, clinicians must carefully assess the risk of cervical lesions. This study provides a reference for further understanding the cervical safety of postmenopausal estrogen use.

## Data Availability

The datasets used and/or analysed during the current study are available from the corresponding author on reasonable request.
